# Percutaneous pinning after prolonged skeletal traction with the hip in a flexed position for unstable slipped capital femoral epiphysis: A case series of 11 patients

**DOI:** 10.1097/MD.0000000000006662

**Published:** 2017-05-12

**Authors:** Masaki Matsushita, Kenichi Mishima, Kohji Iwata, Tadashi Hattori, Naoki Ishiguro, Hiroshi Kitoh

**Affiliations:** a Department of Orthopaedics, Nagoya University Graduate School of Medicine, Nagoya; b Department of Orthopaedics, Aichi Children's Health and Medical Center, Aichi, Japan.

**Keywords:** avascular necrosis, skeletal traction, slipped capital femoral epiphysis, unstable

## Abstract

**Background:**

Unstable slipped capital femoral epiphysis (SCFE) has a relatively high risk of avascular necrosis of the femoral head. Standard treatment for unstable SCFE is still controversial. We reviewed unstable SCFE case series treated with the standardized protocol, which consisted of percutaneous pinning after prolonged skeletal traction.

**Methods:**

Our treatment regimen for unstable SCFE patients included 1 week or more of skeletal traction with the hip in a flexed position of 45 degrees, and subsequent percutaneous pinning after unintentional reduction. Eleven patients were treated in our institution and an affiliated hospital between 2003 and 2013. Sex, age at surgery, body mass index, the presence of hormonal abnormality, duration between onset and surgery, head shaft angle, posterior tilting angle, and modified Harris hip score were investigated from the medical records and radiographs.

**Results:**

There were 8 male and 3 female with an average age of 11.7 years and an average body mass index of 24.5 kg/mm^2^. No patients had hormonal abnormalities. The duration between onset and surgery ranged from 8 to 16 days. The average preoperative and postoperative head shaft angles were 126 and 141 degrees, respectively. Postoperative posterior tilting angle was averaged of 30.7 degrees, which decreased to 21.5 degrees during follow-up period. One patient showed mild avascular necrosis only confirmed by magnetic resonance imaging, but he was uneventfully treated without additional procedures. As a result, all patients had a perfect modified Harris hip score of 91 points.

**Conclusions:**

Prolonged traction with the hip in a flexed position may not only provide gradual reduction of posteriorly displaced epiphysis but also decrease intra-articular pressure before surgery. Although percutaneous pinning after unintentional reduction leaves mild displacement of the femoral epiphysis, remodeling could be expected during remaining growth period.

## 1 Introduction

Unstable slipped capital femoral epiphysis (SCFE) has a significant risk of avascular necrosis (AVN) of the femoral head.^[1]^ The exact etiology of AVN has not been determined, but potential mechanisms for the development of AVN may partially depend on the type of treatment.^[2]^ Recently, favorable clinical outcome with a low incidence of AVN was demonstrated by the modified Dunn procedure.^[3–5]^ On the contrary, closed reduction and screw fixation have shown a relatively higher rate of AVN, ranging from 20% to 50%.^[6]^ Parsch et al^[7]^ reported a low AVN rate (<10%) by controlled gentle reduction and K-wire fixation associated with capsulotomy for evacuation of intra-articular effusion or hematoma. Safe and reliable treatment for unstable SCFE, however, has not been established.

There are some reports showing that the duration from onset of the disease to surgical intervention correlated to the occurrence of AVN. The period from 24 hours to 7 days after onset is accepted to be “unsafe window,” which shows a higher risk of ANV.^[8,9]^ Several investigators have recommended an urgent (before 24 hours) surgical reduction to relieve the pressure and allow reperfusion of the femoral head.^[10]^ Others recommend that definitive stabilization should be delayed for at least 7 days to allow the inflammation and synovitis to settle.^[11,12]^ Vegter^[13]^ proposed that increased intra-articular pressure of the hip associated with excess joint fluid should be treated by traction in a flexed position until the effusion settles. To decrease intra-articular pressure and prevent the occurrence of AVN, we applied skeletal traction with the hip in a flexed position during the acute phase (more than 7 days) for unstable SCFE.

We retrospectively reviewed clinical outcome of unstable SCFE patients who were treated with the standardized protocol in our institution and an affiliated hospital between 2003 and 2013. Our protocol included 1 week or more of preoperative skeletal traction with an affected hip in a flexed position and subsequent percutaneous pinning after unintentional reduction. We found less femoral head deformity with a low rate of AVN in our case series. We propose our technique could be 1 of the therapeutic options for the treatment of unstable SCFE.

## 2 Patients and methods

We retrospectively reviewed the medical records and radiographs of all SCFE patients who were surgically treated at our institution and an affiliated hospital between 2003 and 2013 with the institutional review board approval of Nagoya University Hospital. Unstable SCFE was determined according to the report of Loder et al.^[1]^ Inclusion criteria were the unstable SCFE patients who had adequate clinical, radiological, and operative documentation, and were followed at least 1 year after surgery.

We investigated the age at onset, the body mass index (BMI), the presence of hormonal abnormalities, and the duration between onset and surgery. The head shaft angle (HSA) and the posterior tilting angle (PTA) were measured based on anteroposterior and Lauenstein radiographs of the hip, respectively. Preoperative PTA could not been obtained in some patients because it was too difficult to keep Lauenstein position due to their severe hip pain. Postoperative HSA was measured by the radiographs taken within 1 month after the surgery. The PTA was evaluated postoperatively (within 1 month after surgery) and at the latest follow-up. We investigated the range of motion (ROM) of the hip, and clinical outcome was evaluated based on the modified Harris hip score (mHHS) at the latest follow-up.

Our treatment regimen for unstable SCFE patients had been standardized as follows. Immediately after the diagnosis of unstable SCFE, a Kirscher wire was inserted in the distal femur just proximal to the growth plate under local anesthesia. Then, skeletal traction was commenced with an affected hip in a flexed position of approximately 45 degrees to obtain gradual reduction and reduce intra-articular pressure as much as possible. After more than 1 week of traction period, the patient was transferred to the operating room and fixed gently in a traction table without intensive traction under general anesthesia. Epiphysiodesis was, then, performed using 2 cannulated SCFE screws (DePuy Synthes, Tokyo, Japan) to obtain firm fixation against the unstable epiphysis. No postoperative immobilization was encouraged, but weight-bearing was prohibited for a minimal period of 3 months after surgery. After confirming that there was no presence of AVN by magnetic resonance imaging (MRI), and also radiographs at 3 months after surgery, partial weight-bearing was allowed. Full weight-bearing was commenced at 6 months after surgery. Nonweight-bearing period was extended when the AVN was found.

## 3 Results

**Figure 1:**
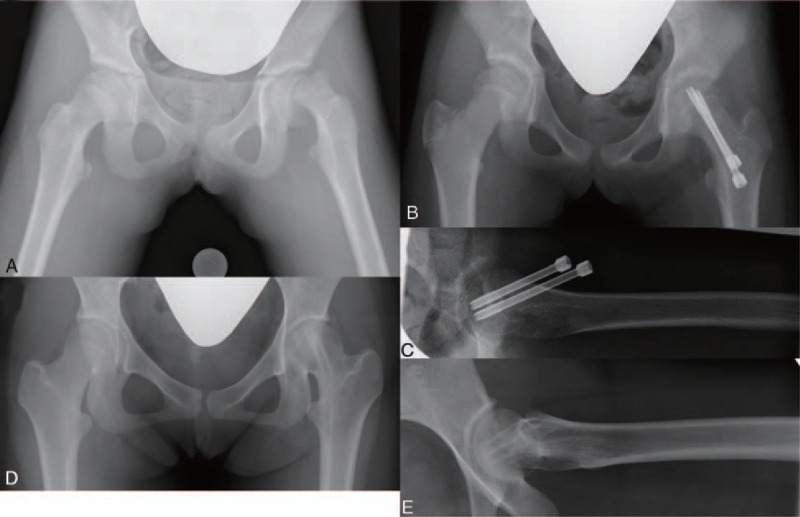
Preoperative anteroposterior hip radiograph of case 6 (A) demonstrating severely displaced femoral epiphysis with the HSA of 104 degrees. Postoperative anteroposterior (B) and Lauenstein (C) hip radiographs revealing reduced femoral epiphysis with the HSA of 146 degrees and the PTA of 33 degrees. Anteroposterior (D) and Lauenstein (E) hip radiographs at the age of 14 years demonstrating spherical femoral head with no evidence of avascular necrosis. HSA = head shaft angle, PTA = posterior tilting angle.

Details of unstable SCFE patients in the current study were shown in Table 1. There were 8 boys and 3 girls with an average age at surgery of 11.7 years old (range 8.8–14.2 years old) and an average BMI of 24.5 kg/mm^2^ (range 13.8–35.5 kg/mm^2^). No patient had hormonal abnormalities in this study. The average duration between onset and surgery was 11.5 days (range 8–16 days). The average HSA was 126 degrees (range 104–146 degrees) preoperatively and 141 degrees (range 117–154 degrees) postoperatively. The average postoperative and the latest PTA were 30.7 degrees (range 19–43 degrees) and 21.5 degrees (range 9–35 degrees), respectively. Neither pain nor significant limited ROM of the hip joint was observed in patients at the time of final follow-up (Table 2). All patients had a perfect score of mHHS with 2.8 years of average follow-up period (Fig. 1).

**Table 1:**
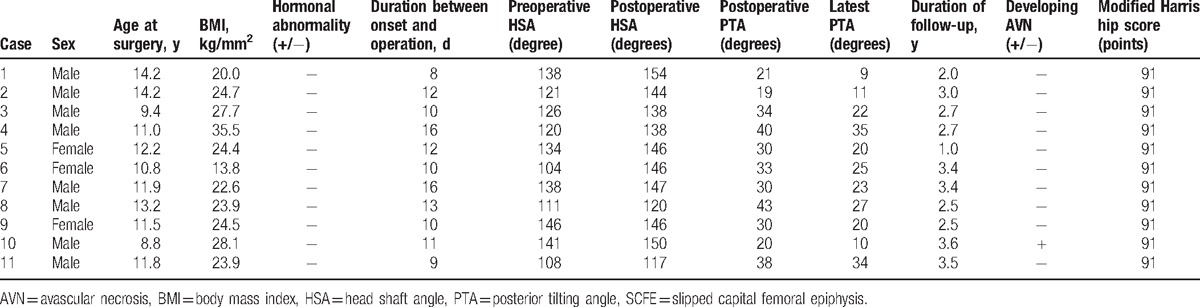
Details of unstable SCFE patients.

**Table 2:**
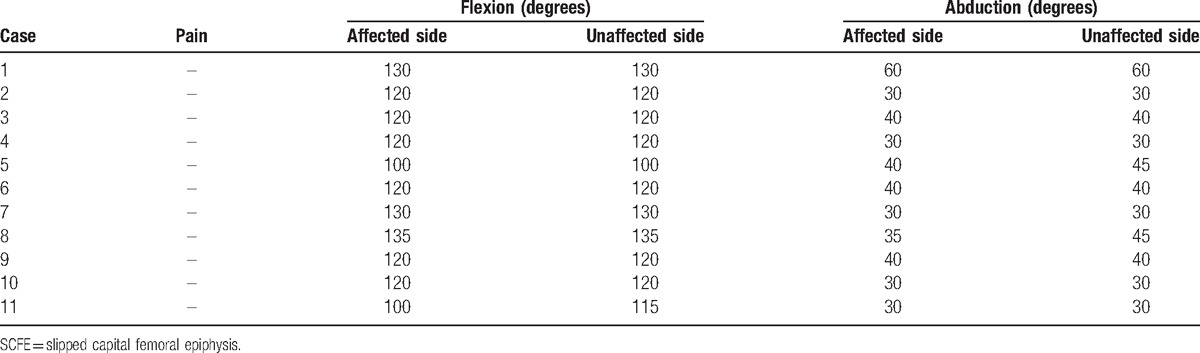
Pain and range of motion at the latest follow-up.

**Figure 2:**
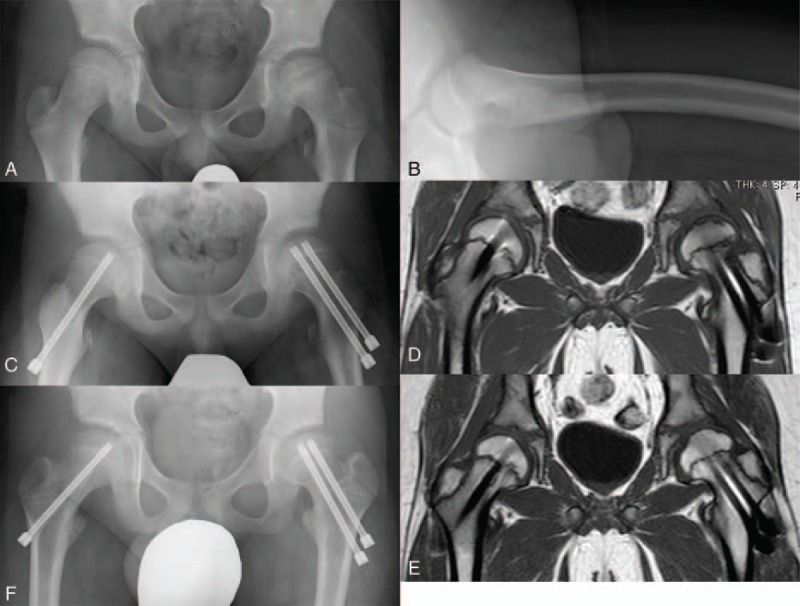
Anteroposterior (A) and Lauenstein (B) hip radiographs of case 10 demonstrating posteriorly displaced femoral epiphysis with the HSA of 141 degrees and the PTA of 35 degrees. Postoperative anteroposterior hip radiograph showing reduced femoral epiphysis of the affected side and prophylactic pinning on the contralateral side (C). Coronal T1-weighted magnetic resonance imaging (MRI) of the hip taken at 3 months after surgery indicating linear low-intensity area at the subchondral bone region of the affected hip (D). Coronal T1-weighted MRI of the hip taken at 1 year after surgery demonstrating disappearance of the abnormal signal intensity observed in previous films (E). Anteroposterior hip radiograph (F) at the age of 10.8 years showing spherical femoral head with no evidence of collapse. HSA = head shaft angle, PTA = posterior tilting angle.

We observed AVN in 1 patient. He showed an uneventful postoperative clinical course, but tiny linear low-intensity area at the subchondral region was observed on the T1 and T2-weighted MRI scan routinely taken at 3 months after surgery (Fig. 2), suggesting the possibility of localized AVN of the femoral head at the weight-bearing surface. We thus continued nonweight-bearing treatment until the confirmation of disappearance of the abnormal finding on MRI. Partial and full weight-bearing were allowed at 1.3 and 1.5 years after surgery, respectively, and he gained full activity without pain and limitation of ROM of the hip joint at the latest follow-up.

## 4 Discussion

We have shown a favorable outcome of the unstable SCFE patients treated with prolonged skeletal traction with an affected hip in a flexed position and subsequent percutaneous pinning after unintentional reduction position. This technique is easy and minimally invasive with a low incidence of AVN compared with open surgeries such as the Dunn procedure.

The posterior-superior retinacular vessels provide the major blood supply to the epiphysis of the femoral head.^[14]^ Kinking or twisting of these blood vessels may compromise the blood flow to the femoral epiphysis in a traumatic circumstance, as in unstable SCFE. Gradual hip traction in a flexed position could facilitate a reduction of the posteriorly displaced femoral epiphysis atraumatically, and relieve the kinking or twisting of these vessels.

An inflammatory effusion and increased intra-articular pressure associated with an unstable SCFE might further compromise an already tenuous blood supply.^[15]^ Therefore, it is important to reduce the intra-articular pressure to prevent the development of AVN in unstable SCFE. Vegter^[13]^ investigated the intra-articular pressure in patients with transient synovitis and Perthes disease, and demonstrated that extension and internal rotation caused an increase in intra-articular pressure which may compromise the blood supply to the capital epiphysis of the femur. The human cadaver study indicated that intra-articular pressure of the hip joint depended mainly on the position of the joint, and it showed the lowest in 45 degrees of flexion.^[16]^ We believe that gradual traction with the affected hip in a flexed position of 45 degrees contributed to prevention of occurrence of AVN by reducing intra-articular pressure in the current study.

Jones et al^[17]^ suggested that in situ pinning may result in impingement of moderate and severe (more than 30 degrees of Southwick angle) acute SCFE. The average final PTA was 21.5 degrees in the current cases, whereas postoperative PTA was 30.7 degrees. Even after stabilization of the slipped epiphysis, femoral neck growth and remodeling were seen radiologically,^[18]^ and also histologically.^[19]^ Actually, there were no patients who showed femoroacetabular impingement in the current study. Even if the mild slip remained just after the surgery, femoroacetabular impingement could be avoidable through the remodeling of femoral head during the residual growth.^[17,18]^

Prolonged skeletal traction with the hip in a flexed position and subsequent screw fixation with an unintentional reduction is a novel and easy technique with a low incidence of complications for the treatment of unstable SCFE. Prospective multicenter studies are needed to determine if the current procedure can maintain the proximal femoral morphology without AVN and delay the onset and progression of osteoarthritis.
